# Supplementary material for: The Hidden Pandemic of COVID-19-Induced Organizing Pneumonia
Source: Pharmaceuticals (Basel). 2022 Dec 16;15(12):1574. doi: 10.3390/ph15121574 (PMC9780828; doi:10.3390/ph15121574)
Supplement: Supplementary file 1 [file pharmaceuticals-15-01574-s001.zip › pharmaceuticals-2052675-supplementary.pdf]

Supplementary Materials:

Table S1. Time dependent Epidemiology of PCOP.

**Reference**			**Acute COVID**				**post-COVID**		**Prevalence, %**				
**Reference**	**Type**	**Risk of bias score**	**Time period**		**Country**	**Severity**	**Sample size, pts**	**Follow-up days**	**DLCO < LLN**	**Dyspnea**	**Cough**	**CT abnormalities**	
			**start**	**end**								**All**	**GGO | Consolidation | Reticulation**
[1]	Letter	8	02/20	03/20	CN	moderate	24	34	30.4	-	-	-	
[2]	Letter	7	03/20	04/20	FR	moderate-to-critical	50	30	-	-	-	66.0	
[3]	Article	8	01/20	03/20	CN	severe	40	30	42.5	-	-	37.5	
[1]	Letter	8	02/20	03/20	CN	moderate-to-critical	110	34	47.2	-	-	-	
[1]	Letter	8	02/20	03/20	CN	severe	67	34	42.4	-	-	-	
[4]	Article	7	02/20	05/20	UK	moderate-to-critical	77	42	-	-	-	76.6	
[5]	Article	7	03/20	04/20	NL	severe	28	42	55.6			-	
[3]	Article	8	01/20	03/20	CN	severe-to-critical	57	30	52.6	7.0	10.5	54.4	
[5]	Article	7	03/20	04/20	NL	severe-to-critical	101	42	71.7	23.8		-	
[6]	Article	7	02/20	07/20	NL	severe-to-critical	92	42	63.0	-	-	78.7	
[3]	Article	8	01/20	03/20	CN	critical	17	30	76.5	-	-	94.1	
[1]	Letter	8	02/20	03/20	CN	critical	19	34	84.2	-	-	-	
[5]	Article	7	03/20	04/20	NL	critical	73	42	78.5		-	-	

<table>
<tr><th colspan="3">Reference</th><th colspan="4">Acute COVID</th><th colspan="2">post-COVID</th><th colspan="5">Prevalence, %</th></tr>
<tr><th rowspan="2">Reference</th><th rowspan="2">Type</th><th rowspan="2">Risk of bias score</th><th colspan="2">Time period</th><th rowspan="2">Country</th><th rowspan="2">Severity</th><th rowspan="2">Sample size, pts</th><th rowspan="2">Follow-up days</th><th rowspan="2">DLCO < LLN</th><th rowspan="2">Dyspnea</th><th rowspan="2">Cough</th><th colspan="2">CT abnormalities</th></tr>
<tr><th>start</th><th>end</th><th>All</th><th>GGO | Consolidation | Reticulation</th></tr>
<tr><td>[7]</td><td>Article</td><td>7</td><td>01/20</td><td>02/20</td><td>CN</td><td>moderate</td><td>41</td><td>87</td><td>42.0</td><td>7.0</td><td>-</td><td>63.8</td><td></td></tr>
<tr><td>[8]</td><td>Article</td><td>8</td><td>02/20</td><td>04/20</td><td>CN</td><td>moderate</td><td>63</td><td>90</td><td>-</td><td>0.0</td><td>7.9</td><td>49.2</td><td>[1.6 | - | -]</td></tr>
<tr><td>[9]</td><td>Article</td><td>9</td><td>01/20</td><td>02/20</td><td>CN</td><td>moderate-to-severe</td><td>55</td><td>93</td><td>16.4</td><td>14.6</td><td>1.8</td><td>71.9</td><td>[7.3 | - | -]</td></tr>
<tr><td>[10]</td><td>Letter</td><td>8</td><td>03/20</td><td>04/20</td><td>ES</td><td>moderate-to-severe</td><td>172</td><td>102</td><td>57.0</td><td>55.0</td><td>18.0</td><td>54.4</td><td></td></tr>
<tr><td>[11]</td><td>Article</td><td>7</td><td>02/20</td><td>08/20</td><td>FR</td><td>moderate-to-critical</td><td>137</td><td>90</td><td>43.0</td><td>-</td><td>-</td><td>75.0</td><td>[75.0 | - | 30.0]</td></tr>
<tr><td>[12]</td><td>Article</td><td>7</td><td>02/20</td><td>03/20</td><td>CN</td><td>moderate-to-critical</td><td>83</td><td>90</td><td>55.0</td><td>81.0</td><td>-</td><td>78.0</td><td>[78.0 | - | 33.0]</td></tr>
<tr><td>[13]</td><td>Article</td><td>8</td><td>03/20</td><td>06/20</td><td>UK</td><td>moderate-to-critical</td><td>80</td><td>105</td><td>-</td><td>46.0</td><td>21.0</td><td>56.0</td><td>[47.9 | 6.8 | 14.0]</td></tr>
<tr><td>[8]</td><td>Article</td><td>8</td><td>02/20</td><td>04/20</td><td>CN</td><td>severe</td><td>378</td><td>90</td><td>-</td><td>3.7</td><td>7.9</td><td>36.8</td><td>[.5 | - | -]</td></tr>
<tr><td>[14]</td><td>Article</td><td>8</td><td>03/20</td><td>05/20</td><td>UK</td><td>severe-to-critical</td><td>58</td><td>69</td><td>-</td><td>64.3</td><td>60.3</td><td>-</td><td></td></tr>
<tr><td>[15]</td><td>Article</td><td>8</td><td>03/20</td><td>06/20</td><td>IR</td><td>severe-to-critical</td><td>101</td><td>82</td><td>-</td><td>-</td><td>-</td><td>72.0</td><td></td></tr>
<tr><td>[7]</td><td>Article</td><td>7</td><td>01/20</td><td>02/20</td><td>CN</td><td>severe-to-critical</td><td>40</td><td>87</td><td>68.0</td><td>23.0</td><td>-</td><td>72.0</td><td></td></tr>
<tr><td>[6]</td><td>Article</td><td>7</td><td>02/20</td><td>07/20</td><td>NL</td><td>severe-to-critical</td><td>92</td><td>90</td><td>51.0</td><td>-</td><td>-</td><td>62.0</td><td>[57.5 | 14.9 | 33.3]</td></tr>
<tr><td>[16]</td><td>Article</td><td>8</td><td>03/20</td><td>06/20</td><td>CA</td><td>severe-to-critical</td><td>73</td><td>91</td><td>59.0</td><td>14.0</td><td>-</td><td>-</td><td></td></tr>
<tr><td>[17]</td><td>Article</td><td>8</td><td>05/20</td><td>07/20</td><td>ES</td><td>severe-to-critical</td><td>313</td><td>63</td><td>54.6</td><td>51.1</td><td>-</td><td>-</td><td></td></tr>
<tr><td>[18]</td><td>Article</td><td>7</td><td>03/20</td><td>08/20</td><td>ES</td><td>critical</td><td>94</td><td>90</td><td>82.0</td><td>-</td><td>-</td><td>56.6</td><td>[29.3 | 17.2 | 43.4]</td></tr>
<tr><td>[8]</td><td>Article</td><td>8</td><td>02/20</td><td>04/20</td><td>CN</td><td>critical</td><td>61</td><td>90</td><td>-</td><td>4.9</td><td>8.2</td><td>45.9</td><td>[1.6 | - | -]</td></tr>
</table>

<table>
<tr><th colspan="3">Reference</th><th colspan="4">Acute COVID</th><th colspan="2">post-COVID</th><th colspan="5">Prevalence, %</th></tr>
<tr><th rowspan="2">Reference</th><th rowspan="2">Type</th><th rowspan="2">Risk of bias score</th><th colspan="2">Time period</th><th rowspan="2">Country</th><th rowspan="2">Severity</th><th rowspan="2">Sample size, pts</th><th rowspan="2">Follow-up days</th><th rowspan="2">DLCO < LLN</th><th rowspan="2">Dyspnea</th><th rowspan="2">Cough</th><th colspan="2">CT abnormalities</th></tr>
<tr><th>start</th><th>end</th><th>All</th><th>GGO | Consolidation | Reticulation</th></tr>
<tr><td>[19]</td><td>Article</td><td>7</td><td>01/20</td><td>09/20</td><td>CN</td><td>moderate</td><td>31</td><td>180</td><td>-</td><td>-</td><td>-</td><td>3.4</td><td>[3.0 | - | .0]</td></tr>
<tr><td>[20]</td><td>Article</td><td>8</td><td>01/20</td><td>05/20</td><td>CN</td><td>moderate</td><td>439</td><td>186</td><td>22.0</td><td>26.0</td><td>-</td><td>52.0</td><td>[41.0 | .0 | .0]</td></tr>
<tr><td>[21]</td><td>Article</td><td>8</td><td>01/20</td><td>05/20</td><td>CN</td><td>moderate</td><td>318</td><td>185</td><td>21.0</td><td>25.0</td><td>-</td><td>100.0</td><td>[85.0 | .0 | .0]</td></tr>
<tr><td>[19]</td><td>Article</td><td>7</td><td>01/20</td><td>09/20</td><td>CN</td><td>moderate-to-severe</td><td>54</td><td>180</td><td>32.1</td><td>18.5</td><td>5.6</td><td>-</td><td></td></tr>
<tr><td>[22]</td><td>Article</td><td>6</td><td>01/20</td><td>10/20</td><td>CN</td><td>moderate-to-critical</td><td>50</td><td>180</td><td>-</td><td>2.0</td><td>10.0</td><td>91.0</td><td>[42.0 | 20.0 | 11.0]</td></tr>
<tr><td>[12]</td><td>Article</td><td>7</td><td>02/20</td><td>03/20</td><td>CN</td><td>moderate-to-critical</td><td>83</td><td>180</td><td>54.0</td><td>30.0</td><td>-</td><td>46.0</td><td>[46.0 | - | 16.0]</td></tr>
<tr><td>[23]</td><td>Article</td><td>6</td><td>12/19</td><td>02/20</td><td>CN</td><td>moderate-to-critical</td><td>114</td><td>175</td><td>26.0</td><td>14.0</td><td>10.0</td><td>26.0</td><td>[62.0 | 24.0 | 14.0]</td></tr>
<tr><td>[24]</td><td>Article</td><td>7</td><td>12/19</td><td>04/20</td><td>CN</td><td>moderate-to-critical</td><td>141</td><td>175</td><td>-</td><td>-</td><td>-</td><td>54.6</td><td>[20.6 | 5.7 | 28.4]</td></tr>
<tr><td>[19]</td><td>Article</td><td>7</td><td>01/20</td><td>09/20</td><td>CN</td><td>severe</td><td>23</td><td>180</td><td>-</td><td>-</td><td>-</td><td>52.6</td><td>[47.0 | - | 16.0]</td></tr>
<tr><td>[25]</td><td>Article</td><td>6</td><td>03/20</td><td>07/20</td><td>TR</td><td>severe</td><td>60</td><td>180</td><td>-</td><td>-</td><td>-</td><td>65.0</td><td></td></tr>
<tr><td>[26]</td><td>Article</td><td>7</td><td>03/20</td><td>05/20</td><td>IT</td><td>moderate-to-critical</td><td>118</td><td>180</td><td>-</td><td>42.0</td><td>24.0</td><td>72.0</td><td>[42.0 | 2.0 | -]</td></tr>
<tr><td>[27]</td><td>Letter</td><td>6</td><td>03/20</td><td>06/20</td><td>IT</td><td>severe</td><td>86</td><td>180</td><td>20.0</td><td></td><td></td><td>.0</td><td></td></tr>
<tr><td>[17]</td><td>Article</td><td>8</td><td>05/20</td><td>07/20</td><td>ES</td><td>severe</td><td>147</td><td>191</td><td></td><td></td><td></td><td>52.4</td><td>[36.7 | - | 10.9]</td></tr>
<tr><td>[28]</td><td>Article</td><td>8</td><td>03/20</td><td>06/20</td><td>IT</td><td>moderate-to-critical</td><td>219</td><td>120</td><td>51.6</td><td>5.5</td><td>2.5</td><td>-</td><td></td></tr>
<tr><td>[20]</td><td>Article</td><td>8</td><td>01/20</td><td>05/20</td><td>CN</td><td>severe</td><td>1172</td><td>186</td><td>29.0</td><td>26.0</td><td>-</td><td>54.0</td><td>[48.0 | 2.0 | 1.0]</td></tr>
<tr><td>[29]</td><td>Article</td><td>6</td><td>03/20</td><td>06/20</td><td>IT</td><td>severe</td><td>71</td><td>180</td><td>58.0</td><td>33.0</td><td>-</td><td>25.0</td><td>[16.0 | .0 | 19.0]</td></tr>
</table>

<table>
<tr><th colspan="3">Reference</th><th colspan="4">Acute COVID</th><th colspan="2">post-COVID</th><th colspan="5">Prevalence, %</th></tr>
<tr><th rowspan="2">Reference</th><th rowspan="2">Type</th><th rowspan="2">Risk of bias score</th><th colspan="2">Time period</th><th rowspan="2">Country</th><th rowspan="2">Severity</th><th rowspan="2">Sample size, pts</th><th rowspan="2">Follow-up days</th><th rowspan="2">DLCO < LLN</th><th rowspan="2">Dyspnea</th><th rowspan="2">Cough</th><th colspan="2">CT abnormalities</th></tr>
<tr><th>start</th><th>end</th><th>All</th><th>GGO | Consolidation | Reticulation</th></tr>
<tr><td>[21]</td><td>Article</td><td>8</td><td>01/20</td><td>05/20</td><td>CN</td><td>severe</td><td>864</td><td>185</td><td>26.0</td><td>25.0</td><td>-</td><td>100.0</td><td>[93.0 | 7.0 | .0]</td></tr>
<tr><td>[30]</td><td>Article</td><td>8</td><td>02/20</td><td>03/20</td><td>CN</td><td>severe-to-critical</td><td>41</td><td>210</td><td>-</td><td>-</td><td>-</td><td>-</td><td>[12.0 | 10.0 | 12.0]</td></tr>
<tr><td>[14]</td><td>Article</td><td>8</td><td>03/20</td><td>05/20</td><td>UK</td><td>severe-to-critical</td><td>46</td><td>190</td><td>52.2</td><td>56.5</td><td>56.5</td><td>-</td><td></td></tr>
<tr><td>[6]</td><td>Article</td><td>7</td><td>02/20</td><td>07/20</td><td>NL</td><td>severe-to-critical</td><td>92</td><td>180</td><td>46.0</td><td>-</td><td>-</td><td>-</td><td>[31.0 | 6.9 | 22.5]</td></tr>
<tr><td>[16]</td><td>Article</td><td>8</td><td>03/20</td><td>06/20</td><td>CA</td><td>severe-to-critical</td><td>73</td><td>189</td><td>46.0</td><td>19.0</td><td>-</td><td>-</td><td>[36.0 | - | -]</td></tr>
<tr><td>[27]</td><td>Letter</td><td>6</td><td>03/20</td><td>06/20</td><td>IT</td><td>severe-to-critical</td><td>135</td><td>180</td><td>34.0</td><td></td><td></td><td>.0</td><td></td></tr>
<tr><td>[17]</td><td>Article</td><td>8</td><td>05/20</td><td>07/20</td><td>ES</td><td>severe-to-critical</td><td>226</td><td>191</td><td>47.0</td><td>35.5</td><td>-</td><td>65.9</td><td>[47.8 | - | 19.0]</td></tr>
<tr><td>[29]</td><td>Article</td><td>6</td><td>03/20</td><td>06/20</td><td>IT</td><td>severe-to-critical</td><td>312</td><td>180</td><td>46.0</td><td>31.0</td><td>-</td><td>24.7</td><td>[9.0 | 2.0 | 19.0]</td></tr>
<tr><td>[18]</td><td>Article</td><td>7</td><td>03/20</td><td>08/20</td><td>ES</td><td>critical</td><td>74</td><td>180</td><td>45.9</td><td>-</td><td>-</td><td>40.7</td><td>[40.7 | 14.8 | 42.0]</td></tr>
<tr><td>[27]</td><td>Letter</td><td>6</td><td>03/20</td><td>06/20</td><td>IT</td><td>critical</td><td>29</td><td>180</td><td>8.0</td><td></td><td></td><td>.0</td><td></td></tr>
<tr><td>[27]</td><td>Letter</td><td>6</td><td>03/20</td><td>06/20</td><td>IT</td><td>critical</td><td>20</td><td>180</td><td>7.0</td><td>-</td><td>-</td><td>-</td><td></td></tr>
<tr><td>[17]</td><td>Article</td><td>8</td><td>05/20</td><td>07/20</td><td>ES</td><td>critical</td><td>79</td><td>191</td><td></td><td></td><td></td><td>91.1</td><td>[68.4 | - | 34.2]</td></tr>
<tr><td>[20]</td><td>Article</td><td>8</td><td>01/20</td><td>05/20</td><td>CN</td><td>critical</td><td>122</td><td>186</td><td>56.0</td><td>36.0</td><td>-</td><td>54.0</td><td>[45.0 | .0 | 1.0]</td></tr>
<tr><td>[29]</td><td>Article</td><td>6</td><td>03/20</td><td>06/20</td><td>IT</td><td>critical</td><td>144</td><td>180</td><td>36.0</td><td>32.0</td><td>-</td><td>24.0</td><td>[7.0 | 1.0 | 19.0]</td></tr>
<tr><td>[29]</td><td>Article</td><td>6</td><td>03/20</td><td>06/20</td><td>IT</td><td>critical</td><td>97</td><td>180</td><td>54.0</td><td>31.0</td><td>-</td><td>44.0</td><td>[12.0 | 8.0 | 34.0]</td></tr>
<tr><td>[21]</td><td>Article</td><td>8</td><td>01/20</td><td>05/20</td><td>CN</td><td>critical</td><td>94</td><td>185</td><td>57.0</td><td>40.0</td><td>-</td><td>100.0</td><td>[82.0 | .0 | 3.0]</td></tr>
</table>

Reference			Acute COVID				post-COVID		Prevalence, %				
Reference	Type	Risk of bias score	Time period		Country	Severity	Sample size, pts	Follow-up days	DLCO < LLN	Dyspnea	Cough	CT abnormalities	
			start	end								All	GGO | Consolidation | Reticulation
[31]	Article	8	01/20	04/20	CN	moderate	104	314.50	24.2	32.7		56.6	[13.3 | - | -]
[32]	Article	9	01/20	02/20	CN	moderate	51	345	20.0	19.6	-	-	[35.3 | .0 | 2.0]
[33]	Article	9	01/20	03/20	CN	moderate	9	365	11.0	-	-	-	
[8]	Article	8	02/20	04/20	CN	moderate	52	365	-	3.8	1.9	36.8	[.0 | - | -]
[21]	Article	8	01/20	05/20	CN	moderate	318	349	23.0	25.0	-	39.0	[39.0 | .0 | .0]
[34]	Article	8	02/20	03/20	CN	moderate-to-severe	118	349	25.7	-	-	47.4	[16.2 | - | -]
[33]	Article	9	01/20	03/20	CN	moderate-to-severe	119	365	39.0	-	-	-	
[12]	Article	7	02/20	03/20	CN	moderate-to-critical	83	270	-	22.0	-	24.0	[24.0 | - | 4.0]
[12]	Article	7	02/20	03/20	CN	moderate-to-critical	83	360	33.0	5.0	-	23.0	[23.0 | - | 4.0]
[13]	Article	8	03/20	06/20	UK	moderate-to-critical	80	364	-	19.0	13.0	19.0	
[24]	Article	7	12/19	04/20	CN	moderate-to-critical	141	351	-	-	-	52.0	
[31]	Article	8	01/20	04/20	CN	severe	16	314.50	37.5	75.0		57.1	[35.7 | - | -]
[32]	Article	9	01/20	02/20	CN	moderate-to-critical	94	345	14.3	23.4	72.3	71.3	[40.4 | 2.1 | 4.3]
[33]	Article	9	01/20	03/20	CN	severe	82	365	38.0	-	-	-	
[8]	Article	8	02/20	04/20	CN	severe	379	365	-	1.8	2.9	36.8	[.0 | - | -]
[21]	Article	8	01/20	05/20	CN	severe	864	349	31.0	31.0	-	40.0	[27.0 | .0 | 2.0]

<table>
<tr><th colspan="3">Reference</th><th colspan="4">Acute COVID</th><th colspan="2">post-COVID</th><th colspan="5">Prevalence, %</th></tr>
<tr><th rowspan="2">Reference</th><th rowspan="2">Type</th><th rowspan="2">Risk of bias score</th><th colspan="2">Time period</th><th rowspan="2">Country</th><th rowspan="2">Severity</th><th rowspan="2">Sample size, pts</th><th rowspan="2">Follow-up days</th><th rowspan="2">DLCO < LLN</th><th rowspan="2">Dyspnea</th><th rowspan="2">Cough</th><th colspan="2">CT abnormalities</th></tr>
<tr><th>start</th><th>end</th><th>All</th><th>GGO | Consolidation | Reticulation</th></tr>
<tr><td>[35]</td><td>Article</td><td>7</td><td>03/20</td><td>06/20</td><td>IT</td><td>severe</td><td>61</td><td>365</td><td>53.0</td><td>43.0</td><td>-</td><td>46.0</td><td>[9.0 | 4.0 | 27.0]</td></tr>
<tr><td>[36]</td><td>Article</td><td>8</td><td>03/20</td><td>06/20</td><td>IT</td><td>moderate-to-critical</td><td>200</td><td>366</td><td>49.0</td><td>8.1</td><td>11.2</td><td>-</td><td></td></tr>
<tr><td>[32]</td><td>Article</td><td>9</td><td>01/20</td><td>02/20</td><td>CN</td><td>severe-to-critical</td><td>43</td><td>345</td><td>8.6</td><td>27.9</td><td>-</td><td>-</td><td>[46.5 | 4.7 | 7.0]</td></tr>
<tr><td>[37]</td><td>Article</td><td>8</td><td>01/20</td><td>03/20</td><td>CN</td><td>severe-to-critical</td><td>209</td><td>365</td><td>-</td><td>-</td><td>-</td><td>25.4</td><td>[24.9 | 10.7 | 19.6]</td></tr>
<tr><td>[18]</td><td>Article</td><td>7</td><td>03/20</td><td>08/20</td><td>ES</td><td>critical</td><td>37</td><td>360</td><td>29.7</td><td>12.8</td><td>-</td><td>48.8</td><td>[65.9 | 7.3 | 53.7]</td></tr>
<tr><td>[33]</td><td>Article</td><td>9</td><td>01/20</td><td>03/20</td><td>CN</td><td>critical</td><td>28</td><td>365</td><td>54.0</td><td>-</td><td>-</td><td>-</td><td></td></tr>
<tr><td>[8]</td><td>Article</td><td>8</td><td>02/20</td><td>04/20</td><td>CN</td><td>critical</td><td>55</td><td>365</td><td>-</td><td>1.8</td><td>1.8</td><td>45.9</td><td>[.0 | - | -]</td></tr>
<tr><td>[21]</td><td>Article</td><td>8</td><td>01/20</td><td>05/20</td><td>CN</td><td>critical</td><td>94</td><td>349</td><td>54.0</td><td>39.0</td><td>-</td><td>87.0</td><td>[76.0 | 3.0 | 8.0]</td></tr>
<tr><td>[35]</td><td>Article</td><td>7</td><td>03/20</td><td>06/20</td><td>IT</td><td>critical</td><td>136</td><td>365</td><td>29.0</td><td>36.0</td><td>-</td><td>65.0</td><td>[28.0 | 3.0 | 32.0]</td></tr>
<tr><td>[35]</td><td>Article</td><td>7</td><td>03/20</td><td>06/20</td><td>IT</td><td>critical</td><td>90</td><td>365</td><td>49.0</td><td>40.0</td><td>-</td><td>80.0</td><td>[24.0 | 2.0 | 49.0]</td></tr>
</table>

The most part of studies was taken from Lee et al. review [38]. Additionally, studies published in 2020-2021 were located using PubMed. The reliability of the studies was assessed according to the Preferred Reporting Items of Systematic reviews and MetaAnalyses (PRISMA) [39]. Studies with scores below 6 were not included, the reliability of studies with scores of 6 was considered as low, 7-8 — medium, and 9-10 — high reliability. Data on the specified variables were extracted from the selected studies. The extracted data included the recruitment period, demographics of the included patients, data on the severity of acute COVID-19; sample size, follow-up interval after acute COVID-19; data on the prevalence of DLCO impairment using a cutoff of 80% of the predicted values or LLN, prevalence of dyspnea and cough; the proportion of patients with any residual abnormal findings on follow-up CT, prevalence of GGO, consolidation and reticulation among these patients. Disease severity was determined according to the current global consensus guidelines and a few modifications per Lee et al. [38,40] due to the lack of universal disease severity criteria. Patients requiring supplemental oxygen therapy were considered as severe, and requiring intensive care unit admission and/or mechanical ventilation were considered as having critical COVID-19. Low, moderate, and high risks of bias are indicated by scores of 9–10, 7–8, and ≤6, respectively [41].
DLCO = diffusing capacity for carbon monoxide, LLN = lower limit of normal, CT = computed tomography, CGO = ground-glass opacity.

References

1. Mo, X.; Jian, W.; Su, Z.; Chen, M.; Peng, H.; Peng, P.; Lei, C.; Chen, R.; Zhong, N.; Li, S. Abnormal pulmonary function in COVID-19 patients at time of hospital discharge. *Eur Respir J* **2020**, *55*, 2001217. 10.1183/13993003.01217-2020.
2. Frija-Masson, J.; Debray, M.-P.; Gilbert, M.; Lescure, F.-X.; Travert, F.; Borie, R.; Khalil, A.; Crestani, B.; d'Ortho, M.-P.; Bancal, C. Functional characteristics of patients with SARS-CoV-2 pneumonia at 30 days post-infection. *Eur Respir J* **2020**, *56*, 2001754. 10.1183/13993003.01754-2020.
3. Huang, Y.; Tan, C.; Wu, J.; Chen, M.; Wang, Z.; Luo, L.; Zhou, X.; Liu, X.; Huang, X.; Yuan, S.; et al. Impact of coronavirus disease 2019 on pulmonary function in early convalescence phase. *Respir Res* **2020**, *21*, 163. 10.1186/s12931-020-01429-6.
4. Myall, K.J.; Mukherjee, B.; Castanheira, A.M.; Lam, J.L.; Benedetti, G.; Mak, S.M.; Preston, R.; Thillai, M.; Dewar, A.; Molyneaux, P.L.; et al. Persistent Post-COVID-19 Interstitial Lung Disease. An Observational Study of Corticosteroid Treatment. *Ann Am Thorac Soc* **2021**, *18*, 799–806. 10.1513/AnnalsATS.202008-1002OC.
5. van der Sar-van der Brugge, S.; Talman, S.; Boonman-de Winter, L.; de Mol, M.; Hoefman, E.; van Etten, R.W.; De Backer, I.C. Pulmonary function and health-related quality of life after COVID-19 pneumonia. *Respir Med* **2021**, *176*, 106272. 10.1016/j.rmed.2020.106272.
6. Hellemons, M.E.; Huijts, S.; Bek, L.M.; Berentschot, J.C.; Nakshbandi, G.; Schurink, C.A.M.; Vlake, J.H.; van Genderen, M.E.; van Bommel, J.; Gommers, D.; et al. Persistent Health Problems beyond Pulmonary Recovery up to 6 Months after Hospitalization for COVID-19: A Longitudinal Study of Respiratory, Physical, and Psychological Outcomes. *Ann Am Thorac Soc* **2022**, *19*, 551–561. 10.1513/AnnalsATS.202103-340OC.
7. Qin, W.; Chen, S.; Zhang, Y.; Dong, F.; Zhang, Z.; Hu, B.; Zhu, Z.; Li, F.; Wang, X.; Wang, Y.; et al. Diffusion capacity abnormalities for carbon monoxide in patients with COVID-19 at 3-month follow-up. *Eur Respir J* **2021**, *58*, 2003677. 10.1183/13993003.03677-2020.
8. Liu, T.; Wu, D.; Yan, W.; Wang, X.; Zhang, X.; Ma, K.; Chen, H.; Zeng, Z.; Qin, Y.; Wang, H.; et al. Twelve-Month Systemic Consequences of Coronavirus Disease 2019 (COVID-19) in Patients Discharged From Hospital: A Prospective Cohort Study in Wuhan, China. *Clin Infect Dis* **2022**, *74*, 1953–1965. 10.1093/cid/ciab703.
9. Zhao, Y.-M.; Shang, Y.-M.; Song, W.-B.; Li, Q.-Q.; Xie, H.; Xu, Q.-F.; Jia, J.-L.; Li, L.-M.; Mao, H.-L.; Zhou, X.-M.; et al. Follow-up study of the pulmonary function and related physiological characteristics of COVID-19 survivors three months after recovery. *EClinicalMedicine* **2020**, *25*, 100463. 10.1016/j.eclinm.2020.100463.
10. Sibila, O.; Albacar, N.; Perea, L.; Faner, R.; Torralba, Y.; Hernandez-Gonzalez, F.; Moisés, J.; Sanchez-Ruano, N.; Sequeira-Aymar, E.; Badia, J.R.; et al. Lung Function sequelae in COVID-19 Patients 3 Months After Hospital Discharge. *Arch Bronconeumol* **2021**, *57*, 59–61. 10.1016/j.arbres.2021.01.036.
11. Frija-Masson, J.; Debray, M.-P.; Boussouar, S.; Khalil, A.; Bancal, C.; Motiejunaite, J.; Galarza-Jimenez, M.A.; Benzaquen, H.; Penaud, D.; Laveneziana, P.; et al. Residual ground glass opacities three months after Covid-19 pneumonia correlate to alteration of respiratory function: The post Covid M3 study. *Respir Med* **2021**, *184*, 106435. 10.1016/j.rmed.2021.106435.
12. Wu, X.; Liu, X.; Zhou, Y.; Yu, H.; Li, R.; Zhan, Q.; Ni, F.; Fang, S.; Lu, Y.; Ding, X.; et al. 3-month, 6-month, 9-month, and 12-month respiratory outcomes in patients following COVID-19-related hospitalisation: a prospective study. *Lancet Respir Med* **2021**, *9*, 747–754. 10.1016/S2213-2600(21)00174-0.
13. Vijayakumar, B.; Tonkin, J.; Devaraj, A.; Philip, K.E.J.; Orton, C.M.; Desai, S.R.; Shah, P.L. CT Lung Abnormalities after COVID-19 at 3 Months and 1 Year after Hospital Discharge. *Radiology* **2022**, *303*, 444–454. 10.1148/radiol.2021211746.
14. Cassar, M.P.; Tunnicliffe, E.M.; Petousi, N.; Lewandowski, A.J.; Xie, C.; Mahmod, M.; Samat, A.H.A.; Evans, R.A.; Brightling, C.E.; Ho, L.-P.; et al. Symptom Persistence Despite Improvement in

Cardiopulmonary Health - Insights from longitudinal CMR, CPET and lung function testing post-COVID-19. *EClinicalMedicine* **2021**, *41*, 101159. 10.1016/j.eclinm.2021.101159.
15. Wallis, T.J.M.; Heiden, E.; Horno, J.; Welham, B.; Burke, H.; Freeman, A.; Dexter, L.; Fazleen, A.; Kong, A.; McQuitty, C.; et al. Risk factors for persistent abnormality on chest radiographs at 12-weeks post hospitalisation with PCR confirmed COVID-19. *Respir Res* **2021**, *22*, 157. 10.1186/s12931-021-01750-8.
16. Shah, A.S.; Ryu, M.H.; Hague, C.J.; Murphy, D.T.; Johnston, J.C.; Ryerson, C.J.; Carlsten, C.; Wong, A.W. Changes in pulmonary function and patient-reported outcomes during COVID-19 recovery: a longitudinal, prospective cohort study. *ERJ Open Res* **2021**, *7*, 00243–02021. 10.1183/23120541.00243-2021.
17. Safont, B.; Tarraso, J.; Rodriguez-Borja, E.; Fernández-Fabrellas, E.; Sancho-Chust, J.N.; Molina, V.; Lopez-Ramirez, C.; Lope-Martinez, A.; Cabanes, L.; Andreu, A.L.; et al. Lung Function, Radiological Findings and Biomarkers of Fibrogenesis in a Cohort of COVID-19 Patients Six Months After Hospital Discharge. *Arch Bronconeumol* **2022**, *58*, 142–149. 10.1016/j.arbres.2021.08.014.
18. González, J.; Zuil, M.; Benítez, I.D.; de Gonzalo-Calvo, D.; Aguilar, M.; Santisteve, S.; Vaca, R.; Minguez, O.; Seck, F.; Torres, G.; et al. One Year Overview and Follow-Up in a Post-COVID Consultation of Critically Ill Patients. *Front Med (Lausanne)* **2022**, *9*, 897990. 10.3389/fmed.2022.897990.
19. Wu, Q.; Zhong, L.; Li, H.; Guo, J.; Li, Y.; Hou, X.; Yang, F.; Xie, Y.; Li, L.; Xing, Z. A Follow-Up Study of Lung Function and Chest Computed Tomography at 6 Months after Discharge in Patients with Coronavirus Disease 2019. *Can Respir J* **2021**, *2021*, 6692409. 10.1155/2021/6692409.
20. Huang, C.; Huang, L.; Wang, Y.; Li, X.; Ren, L.; Gu, X.; Kang, L.; Guo, L.; Liu, M.; Zhou, X.; et al. 6-month consequences of COVID-19 in patients discharged from hospital: a cohort study. *Lancet* **2021**, *397*, 220–232. 10.1016/S0140-6736(20)32656-8.
21. Huang, L.; Yao, Q.; Gu, X.; Wang, Q.; Ren, L.; Wang, Y.; Hu, P.; Guo, L.; Liu, M.; Xu, J.; et al. 1-year outcomes in hospital survivors with COVID-19: a longitudinal cohort study. *Lancet* **2021**, *398*, 747–758. 10.1016/S0140-6736(21)01755-4.
22. Dai, S.; Zhao, B.; Liu, D.; Zhou, Y.; Liu, Y.; Lan, L.; Li, Y.; Luo, W.; Zeng, Y.; Li, W. Follow-Up Study of the Cardiopulmonary and Psychological Outcomes of COVID-19 Survivors Six Months After Discharge in Sichuan, China. *Int J Gen Med* **2021**, *14*, 7207–7217. 10.2147/IJGM.S337604.
23. Han, X.; Fan, Y.; Alwalid, O.; Li, N.; Jia, X.; Yuan, M.; Li, Y.; Cao, Y.; Gu, J.; Wu, H.; et al. Six-month Follow-up Chest CT Findings after Severe COVID-19 Pneumonia. *Radiology* **2021**, *299*, E177–E186. 10.1148/radiol.2021203153.
24. Li, Y.; Han, X.; Huang, J.; Alwalid, O.; Jia, X.; Yuan, M.; Cao, Y.; Shao, G.; Cui, Y.; Liu, J.; et al. Follow-up study of pulmonary sequelae in discharged COVID-19 patients with diabetes or secondary hyperglycemia. *Eur J Radiol* **2021**, *144*, 109997. 10.1016/j.ejrad.2021.109997.
25. Bardakci, M.I.; Ozturk, E.N.; Ozkarafakili, M.A.; Ozkurt, H.; Yanc, U.; Yildiz Sevgi, D. Evaluation of long-term radiological findings, pulmonary functions, and health-related quality of life in survivors of severe COVID-19. *J Med Virol* **2021**, *93*, 5574–5581. 10.1002/jmv.27101.
26. Caruso, D.; Guido, G.; Zerunian, M.; Polidori, T.; Lucertini, E.; Pucciarelli, F.; Polici, M.; Rucci, C.; Bracci, B.; Nicolai, M.; et al. Post-Acute Sequelae of COVID-19 Pneumonia: Six-month Chest CT Follow-up. *Radiology* **2021**, *301*, E396–E405. 10.1148/radiol.2021210834.
27. Milanese, M.; Anselmo, M.; Buscaglia, S.; Garra, L.; Goretti, R.; Parodi, L.; Riccio, G.; Tassara, R.; Gnerre, P. COVID-19 6 months after hospital discharge: pulmonary function impairment and its heterogeneity. *ERJ Open Res* **2021**, *7*, 00196–02021. 10.1183/23120541.00196-2021.
28. Bellan, M.; Soddu, D.; Balbo, P.E.; Baricich, A.; Zeppegno, P.; Avanzi, G.C.; Baldon, G.; Bartolomei, G.; Battaglia, M.; Battistini, S.; et al. Respiratory and Psychophysical Sequelae Among Patients With COVID-19 Four Months After Hospital Discharge. *JAMA Netw Open* **2021**, *4*, e2036142. 10.1001/jamanetworkopen.2020.36142.
29. Faverio, P.; Luppi, F.; Rebora, P.; Busnelli, S.; Stainer, A.; Catalano, M.; Parachini, L.; Monzani, A.; Galimberti, S.; Bini, F.; et al. Six-Month Pulmonary Impairment after Severe COVID-19: A Prospective, Multicentre Follow-Up Study. *Respiration* **2021**, *100*, 1078–1087. 10.1159/000518141.

30. Liu, M.; Lv, F.; Huang, Y.; Xiao, K. Follow-Up Study of the Chest CT Characteristics of COVID-19 Survivors Seven Months After Recovery. *Front Med (Lausanne)* **2021**, *8*, 636298. 10.3389/fmed.2021.636298.
31. Zhou, F.; Tao, M.; Shang, L.; Liu, Y.; Pan, G.; Jin, Y.; Wang, L.; Hu, S.; Li, J.; Zhang, M.; et al. Assessment of Sequelae of COVID-19 Nearly 1 Year After Diagnosis. *Front Med (Lausanne)* **2021**, *8*, 717194. 10.3389/fmed.2021.717194.
32. Zhao, Y.; Yang, C.; An, X.; Xiong, Y.; Shang, Y.; He, J.; Qiu, Y.; Zhang, N.; Huang, L.; Jia, J.; et al. Follow-up study on COVID-19 survivors one year after discharge from hospital. *Int J Infect Dis* **2021**, *112*, 173–182. 10.1016/j.ijid.2021.09.017.
33. Yan, X.; Huang, H.; Wang, C.; Jin, Z.; Zhang, Z.; He, J.; Yin, S.; Fan, M.; Huang, J.; Chen, F.; et al. Follow-up study of pulmonary function among COVID-19 survivors 1 year after recovery. *J Infect* **2021**, *83*, 381–412. 10.1016/j.jinf.2021.05.034.
34. Shang, L.; Wang, L.; Zhou, F.; Li, J.; Liu, Y.; Yang, S. Long-term effects of obesity on COVID-19 patients discharged from hospital. *Immun Inflamm Dis* **2021**, *9*, 1678–1685. 10.1002/iid3.522.
35. Faverio, P.; Luppi, F.; Rebora, P.; D'Andrea, G.; Stainer, A.; Busnelli, S.; Catalano, M.; Modafferi, G.; Franco, G.; Monzani, A.; et al. One-year pulmonary impairment after severe COVID-19: a prospective, multicenter follow-up study. *Respir Res* **2022**, *23*, 65. 10.1186/s12931-022-01994-y.
36. Bellan, M.; Baricich, A.; Patrucco, F.; Zeppegno, P.; Gramaglia, C.; Balbo, P.E.; Carriero, A.; Amico, C.S.; Avanzi, G.C.; Barini, M.; et al. Long-term sequelae are highly prevalent one year after hospitalization for severe COVID-19. *Sci Rep* **2021**, *11*, 22666. 10.1038/s41598-021-01215-4.
37. Pan, F.; Yang, L.; Liang, B.; Ye, T.; Li, L.; Li, L.; Liu, D.; Wang, J.; Hesketh, R.L.; Zheng, C. Chest CT Patterns from Diagnosis to 1 Year of Follow-up in Patients with COVID-19. *Radiology* **2022**, *302*, 709–719. 10.1148/radiol.2021211199.
38. Lee, J.H.; Yim, J.-J.; Park, J. Pulmonary function and chest computed tomography abnormalities 6–12 months after recovery from COVID-19: a systematic review and meta-analysis. *Respir Res* **2022**, *23*, 233. 10.1186/s12931-022-02163-x.
39. Liberati, A.; Altman, D.G.; Tetzlaff, J.; Mulrow, C.; Gotzsche, P.C.; Ioannidis, J.P.A.; Clarke, M.; Devereaux, P.J.; Kleijnen, J.; Moher, D. The PRISMA statement for reporting systematic reviews and meta-analyses of studies that evaluate healthcare interventions: explanation and elaboration. *BMJ* **2009**, *339*, b2700–b2700. 10.1136/bmj.b2700.
40. World Health Organization *COVID-19 clinical management: living guidance, 25 January 2021*; World Health Organization, 2021;
41. Hoy, D.; Brooks, P.; Woolf, A.; Blyth, F.; March, L.; Bain, C.; Baker, P.; Smith, E.; Buchbinder, R. Assessing risk of bias in prevalence studies: modification of an existing tool and evidence of interrater agreement. *J Clin Epidemiol* **2012**, *65*, 934–939. 10.1016/j.jclinepi.2011.11.014.
